# Taming pet allergens: strategies to make furry cats less allergenic

**DOI:** 10.3389/falgy.2026.1930696

**Published:** 2026-08-26

**Authors:** Carole Guillet, Alessia Lüthi, Lara Šošić, Marta Paolucci, Thomas M. Kündig, Pål Johansen

**Affiliations:** 1 Department of Dermatology, University Hospital Zurich, Zurich, Switzerland; 2 Department of Dermatology, University of Zurich, Zurich, Switzerland

**Keywords:** allergy, animal dander, animal fur, cat allergy, cats, environmental control, pets, vaccination

## Abstract

Much research on allergy to furry animals focuses on diagnosis and treatment of the human patients. However, this review will explore strategies aimed at reducing the allergenic potential of the furry animals themselves. A primary focus is vaccination of cats to induce allergen-specific IgG and IgA antibodies. These antibodies can bind the major allergens, potentially prevent their shedding, or neutralise them when exposure occurs. By consequence, neutralising antibodies can reduce IgE-mediated reactions in humans. Two key studies by Thoms et al. (PMID: 32155887; 31056187) provide proof-of-concept for this approach. After vaccination of cats, these produced anti-Fel d 1 IgG and IgA and the cat owners experienced improved tolerance to their pets. Complementary strategies include physical removal of allergens from furry animals through grooming methods such as combing, bathing, or using wipes and enzymatic washing agents, which can reduce the allergen load in the environment. Finally, selective breeding and choice of animal breeds with inherently lower allergen production or reduced shedding may further mitigate exposure. Together, these interventions represent a shift from exclusively human-targeted allergy therapies toward integrated approaches that manage allergen sources at their origin. This review will discuss current evidence, practical implications, and future directions for allergen-reducing strategies in furry animals.

## Introduction: a paradigm shift in cat allergy management

Pet animals play an increasingly important role in modern society (1–3). Dogs and cats are among the most owned pets worldwide and provide substantial emotional, psychological, and social benefits to their owners (4–6). However, exposure to pet allergens is also a major cause of allergic disease, affecting a significant proportion of the population (4), and contributing to the global burden of allergic rhinitis and asthma (7). As rates of pet ownership continue to rise (8), the number of individuals exposed to pet allergens is likely to increase accordingly.

Pet allergy is associated with a broad spectrum of clinical manifestations, ranging from rhinoconjunctivitis and skin symptoms to asthma and severe exacerbations in sensitised individuals (7, 9). Cat and dog allergens are among the most important indoor allergens, and sensitisation to furry animals has been associated with increased asthma prevalence, greater disease severity, and impaired quality of life (4, 10). Importantly, pet allergens are not confined to pet-owning households but are widely distributed in public and private indoor environments (11), making complete avoidance difficult even for individuals who do not own pets (12, 13).

The development and severity of pet allergy are influenced by multiple interacting factors, including allergen exposure, epithelial barrier integrity, host immune responses, and environmental determinants. Rather than representing a single disease mechanism, allergic sensitization results from complex interactions between the animal, the environment, and the allergic individual. Recent research have highlighted the importance of these mechanisms and emphasized the need for integrated approaches that address both allergen exposure and host susceptibility (14, 15). Within this broader framework, source-directed strategies aim to reduce allergen burden at its origin and complement established patient-directed interventions.

Current management relies largely on allergen avoidance, pharmacotherapy and allergen immunotherapy (AIT), each with important limitations. Avoidance is often impractical because pet allergens are ubiquitous, and many patients are unwilling to part with their companion animals. Pharmacological treatments can effectively control symptoms but do not modify the underlying allergic disease. Although AIT remains the only disease-modifying treatment currently available, its role in pet allergy remains controversial (5, 16). Compared with pollen or venom immunotherapy, the evidence base supporting cat and dog AIT is more limited, treatment responses appear more variable, and safety concerns may arise in patients with concomitant asthma (16–19) Important questions remain regarding the efficacy, safety, patient selection, and optimal implementation of pet AIT (5). Consequently, there remains a need for both improved source-directed allergen reduction strategies and safer, more effective patient-directed immunomodulatory therapies. Approaches for safer pet AIT have reported with the production of hypoallergenic allergen derivates that cause less allergic adverse events when applied in conventional subcutaneous immunotherapy (SCIT) (20) or by using intralymphatic immunotherapy (ILIT) that requires less injections with lower allergen doses (21).

The challenges associated with pet allergy are further complicated by the strong emotional bond between humans and their pets. For many individuals and families, pet ownership provides substantial benefits that make pet removal undesirable or unacceptable. Thus, the goal of allergy management extends beyond symptom control alone and increasingly encompasses the preservation of the human-animal relationship, while maintaining the health and well-being of both the patient and the animal.

**Figure 1:**
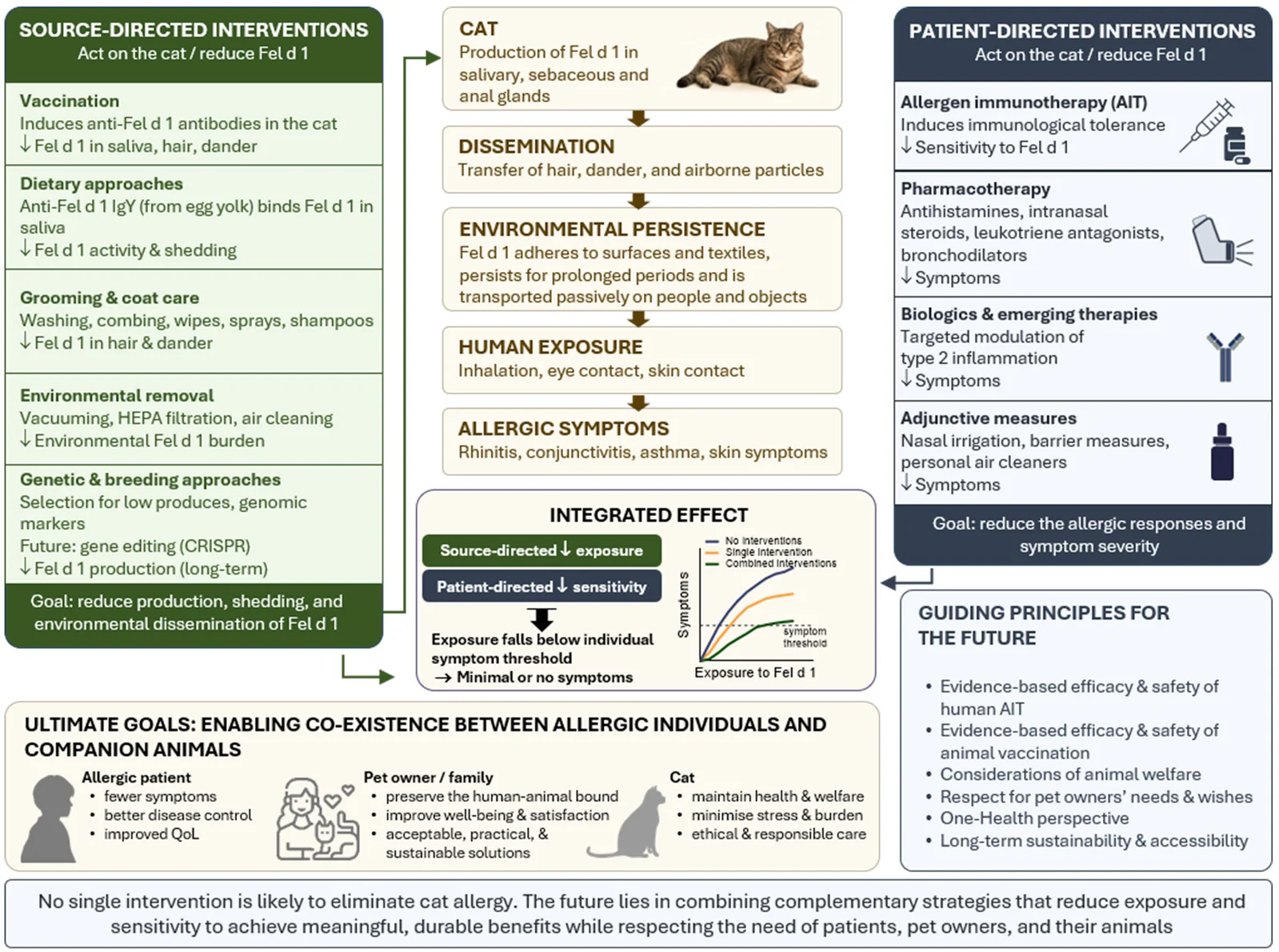
Conceptual overview of integrated management strategies for cat allergy. Fel d 1 is produced by the cat and subsequently disseminated via the fur into the environment, resulting in human exposure and the development of allergic symptoms in sensitized individuals. Source-directed interventions act at different stages of the allergen pathway, including allergen production (vaccination, genetic approaches), allergen activity (vaccination-induced antibodies and dietary anti-Fel d 1 IgY), allergen deposition on the fur (grooming, wipes, bathing), and environmental allergen burden (cleaning and air filtration). In parallel, patient-directed interventions, such as allergen immunotherapy, biologics, and pharmacotherapy, aim to reduce host sensitivity and control symptoms. Rather than relying on a single intervention, future management of cat allergy may combine multiple complementary approaches that simultaneously reduce allergen exposure and increase allergen tolerance. The figure illustrates the conceptual objective of integrating management strategies: to lower allergen exposure and host sensitivity below the threshold required to trigger clinically relevant symptoms, thereby improving quality of life, preserving the human–animal bond, and maintaining animal welfare.

Historically, efforts to manage pet allergy have focused almost exclusively on the allergic individual. In recent years, however, a new paradigm has emerged in which the animal itself becomes the target of intervention (4). Advances in vaccination, dietary allergen neutralisation, grooming strategies, and genetic approaches have created opportunities to reduce allergen production, activity, and dissemination before exposure occurs. These source-directed approaches complement conventional patient-directed therapies and may ultimately enable more effective and sustainable management of pet allergy. Below, we discuss such current and emerging strategies, which all aim at reducing the allergenic potential of furry animals. With a particular focus on cats, we examine interventions targeting allergen production, allergen activity, and allergen shedding, and consider how these approaches may be integrated with established patient-directed therapies to reduce disease burden, improve quality of life, preserve the human–animal bond, and ensure animal welfare. Figure 1 and Table 1 illustrate and list overviews of the integrated management strategies for cat allergy.

**Table 1:** Framework for integrated management of cat allergy.

Stage	Example intervention	Primary objective
Allergen production	Vaccination, genetic selection	Reduce allergen generation and biologically active allergen
Allergen activity	Vaccination-induced antibodies, dietary anti-Fel d 1 IgY	Reduce biologically active allergen
Allergen on fur	Grooming, wipes, sprays, bathing	Reduce surface allergen burden
Environmental burden	Cleaning, HEPA filtration	Reduce environmental exposure
Human immune response	Allergen immunotherapy, biologics, pharmacotherapy	Increase tolerance and control symptoms

## Fel d 1 as the primary target

### Biology of Fel d 1

Fel d 1 is the major cat allergen and the principal cause of sensitisation and allergic symptoms in cat-allergic individuals (22, 23). Nearly 95% of cat-allergic patients have specific IgE antibodies directed against Fel d 1, making it the dominant target of the allergic immune response. Unlike many other mammalian allergens, Fel d 1 is produced almost exclusively by cats and therefore represents a highly specific marker of cat allergen exposure. Structurally, Fel d 1 is a secretoglobin consisting of two distinct polypeptide chains linked by three disulfide bonds to form a heterodimer (24–26) Two heterodimers can further associate through non-covalent interactions to form a tetrameric complex (25, 27). Although its precise biological function remains uncertain, Fel d 1 has been proposed to contribute to pheromone transport and chemical communication between cats. Its localisation in sebaceous, salivary, and anal glands (28) suggests a role in the production or dissemination of chemical signals involved in territorial and social behaviours. During grooming, saliva containing Fel d 1 is deposited onto the fur and skin, from where the allergen becomes airborne and is continuously released into the environment.

### Why target Fel d 1?

Several characteristics make Fel d 1 an attractive target for source-directed interventions. First, it is the dominant cat allergen recognised by the vast majority of cat-allergic individuals (29). Seven other and minor allergens (Fel d 2–8) have been described (4, 22, 30). Reducing exposure to Fel d 1 is therefore expected to benefit a large proportion of patients, whereas targeting minor allergens would likely have a more limited impact. Second, production of Fel d 1 is almost exclusively by the cat and the allergen is released into the environment before human exposure occurs. This creates a unique opportunity to intervene at the source rather than solely treating the downstream allergic response in sensitised individuals. Current management strategies—including allergen avoidance, pharmacotherapy, and AIT—are directed at the patient and do not modify allergen production by the animal. In contrast, interventions targeting Fel d 1 production, activity, or shedding have the potential to reduce allergen exposure for all individuals interacting with the cat. Third, Fel d 1 possesses several characteristics that make it amenable to source-directed interventions. Because the allergen is produced by a limited number of tissues, deposited on the fur during grooming, and subsequently disseminated into the environment, multiple opportunities exist to reduce its production, neutralise its biological activity, or limit its environmental accumulation. These characteristics have stimulated the development of diverse strategies aimed at lowering exposure to biologically active Fel d 1.

Taken together, the central role of Fel d 1 in cat allergy, its cat-specific origin, and its accessibility to source-directed interventions make Fel d 1 the logical focal point for emerging approaches to allergy management. Beyond treating the consequences of allergen exposure, the strategies also aim to reduce exposure at its source, representing a significant potent in the management of cat allergy.

### Environmental exposure and clinical relevance of Fel d 1

The clinical significance of Fel d 1 is determined not only by its potent allergenicity but also by its remarkable capacity for environmental dissemination and persistence. Following deposition onto the fur, Fel d 1 is continuously released on hair, dander, and microscopic airborne particles that readily disperse throughout indoor environments and adhere to clothing, furniture, carpets, and other surfaces. Consequently, Fel d 1 may remain detectable for months after a cat has been removed from the home (12, 13, 31, 32). In addition to direct exposure in cat-owning households, passive transport of allergen on clothing and personal belongings contributes to the widespread distribution of Fel d 1. Measurable allergen levels have been detected in schools, workplaces, hotels, public transportation, and other environments where cats have never been present (12, 33). Consequently, complete avoidance of cat allergen exposure is often difficult to achieve, even for individuals who do not own cats themselves.

This widespread dissemination explains why allergen avoidance remains one of the greatest challenges in cat allergy management. Exposure to Fel d 1 has been associated with the development and exacerbation of allergic rhinitis, conjunctivitis, and asthma in sensitised individuals (6, 23, 34). Symptoms may occur following direct contact with cats, but clinically relevant exposure can also result from indirect environmental contamination. For many patients, particularly cat owners, avoidance strategies are further complicated by the strong emotional bond between humans and their companion animals, making permanent separation from the pet an undesirable or impractical option.

Traditional recommendations for reducing exposure have focused primarily on environmental control measures, including intensive cleaning (35), high-efficiency particulate air (HEPA) filtration (36), restricting cat access to specific rooms, or removal of the animal from the home. Although these approaches may reduce allergen burden to varying degrees, their effectiveness is often limited by the continuous production and dissemination of Fel d 1. Furthermore, substantial reductions in environmental allergen levels may require prolonged and sustained interventions.

The persistence and widespread dissemination of Fel d 1 highlight a fundamental limitation of conventional allergen avoidance strategies: they act only after the allergen has already been produced and dispersed. As a result, complete allergen avoidance is virtually impossible, even for individuals who do not own cats. These limitations have stimulated the development of source-directed interventions that aim to reduce allergen production, neutralise allergen activity, or limit allergen shedding before widespread environmental dissemination occurs. Rather than attempting to eliminate allergen exposure after the fact, these approaches seek to intervene at the earliest stages of the allergen pathway, forming the conceptual basis of an emerging paradigm in cat allergy management.

## Immunological neutralisation of Fel d 1 through cat vaccination

### Concept and immunological rationale

**Figure 2:**
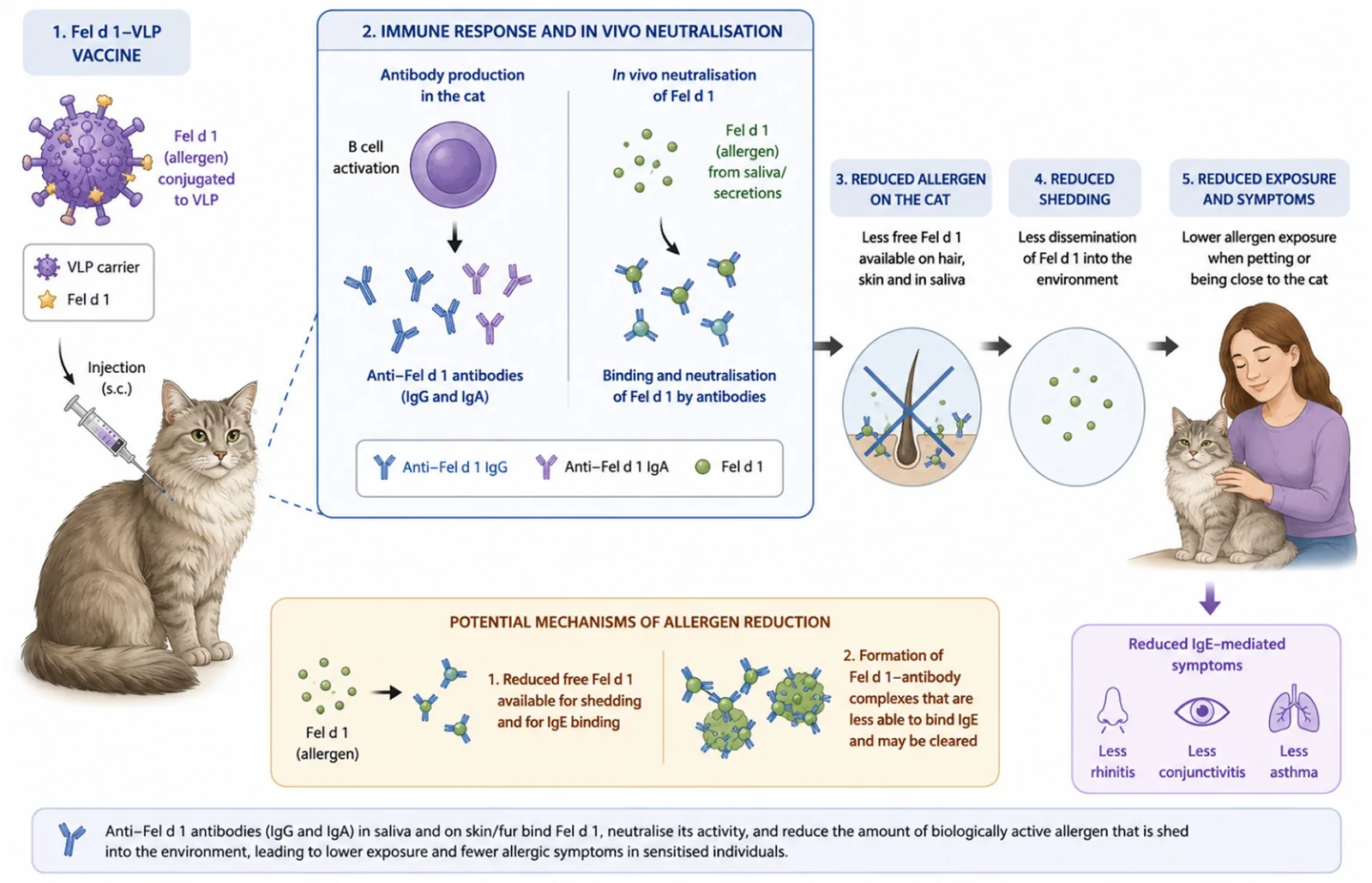
Proposed mechanism of allergen reduction following vaccination of cats against Fel d 1. Following immunisation with a Fel d 1-conjugated VLP vaccine, cats generate Fel d 1-specific antibodies that bind and neutralize the major cat allergen *in vivo*. Antibody-mediated neutralisation may reduce the amount of biologically active Fel d 1 deposited on the coat and released into the environment, thereby lowering allergen exposure in sensitised individuals. This source-directed strategy aims to reduce allergen burden before human exposure occurs and may complement patient-directed approaches such as AIT and pharmacotherapy. Figure was in part adopted and expanded from Thoms et al. (38).

Among the emerging source-directed approaches for managing cat allergy, vaccination of cats against their own major allergen represents an emerging source-directed strategy that aims to reduce the allergenic potential of cats. Rather than treating allergic individuals after exposure has occurred, this approach aims to reduce the amount of biologically active allergen released into the environment by the cat itself. The concept is based on active immunisation against Fel d 1 (37, 38) and illustrated in Figure 2. Following vaccination, cats develop Fel d 1-specific antibodies that bind the allergen before or after secretion. Several mechanisms may contribute to the resulting reduction in allergenic burden. First, antibody binding may reduce the amount of free Fel d 1 available for environmental dissemination. Second, immune complexes formed between Fel d 1 and vaccine-induced antibodies may reduce the ability of the allergen to interact with allergen-specific IgE antibodies in sensitised individuals. Consequently, vaccination has the potential to reduce both allergen exposure and allergenic activity, although the relative contribution of these mechanisms *in vivo* remains incompletely understood.

From an immunological perspective, vaccination against Fel d 1 represents a form of therapeutic self-antigen vaccination, hence, the tolerance to self must be broken. Similar strategies have previously been explored against endogenous targets such as gonadotropin-releasing hormone GnRH (39, 40), nerve growth factor NGF (41–43), PCSK9 (44), and pro-inflammatory cytokines (45, 46). Several studies in horses also have demonstrated the feasibility of stimulating anti-cytokine antibodies through vaccination (47–52) However, Fel d 1 vaccination differs from these approaches in an important respect. Whereas most therapeutic self-antigen vaccines aim to modify disease processes within the vaccinated individual, vaccination against Fel d 1 is primarily intended to benefit another species by reducing allergen exposure in humans while preserving the health and welfare of the cat. In this context, a critical consideration for any self-antigen vaccine is the biological importance of the target molecule. The physiological role of Fel d 1 remains incompletely understood, although functions related to pheromone transport, chemical communication, and social behaviour have been proposed (53). This uncertainty may be viewed both as an opportunity and a challenge. On one hand, the absence of a clearly established essential function and the substantial natural variation in Fel d 1 production among cats suggest that partial neutralisation may be well tolerated. On the other hand, the possibility of currently unrecognised biological functions underscores the importance of continued safety monitoring and long-term follow-up studies.

### Development of a hypocat vaccine

The feasibility of active immunisation against Fel d 1 was demonstrated through the development of a hypocat vaccine platform (37, 38). To overcome immunological tolerance against the self-protein, recombinant Fel d 1 was chemically linked to virus-like particles (VLPs) derived from cucumber mosaic virus. This approach exploits the highly repetitive antigen display and intrinsic immunostimulatory properties of VLPs to induce robust antibody responses against otherwise poorly immunogenic self-antigens. In the initial proof-of-concept study (38), vaccination induced robust anti-Fel d 1 IgG responses in cats. The vaccine was generally well tolerated, and no major vaccine-related adverse effects were reported during the observation period. Importantly, the induced antibodies demonstrated the capacity to bind native Fel d 1, providing evidence that immunological tolerance could be successfully overcome within the duration of the published studies. These findings established the feasibility of therapeutic vaccination against Fel d 1 and provided the foundation for subsequent investigations assessing its impact on allergen activity and human exposure.

### Evidence for allergen reduction and clinical relevance

Subsequent studies extended these observations by evaluating the functional consequences of vaccine-induced anti-Fel d 1 antibodies (37). Vaccination was associated with reduced levels of biologically active Fel d 1 as assessed by inhibition of IgE binding and related surrogate assays (Figure 2). These findings support the central hypothesis that vaccination can reduce the allergenic potential of Fel d 1 without necessarily eliminating its production. Importantly, the concept differs from conventional allergen avoidance measures. Whereas grooming, bathing, or environmental cleaning aim to remove allergen after it has been produced and disseminated, vaccination acts upstream by modifying the allergen before widespread environmental release occurs. As a result, vaccination has the potential to provide sustained reductions in allergen burden while avoiding the need for frequent repetitive interventions.

Despite these encouraging findings, clinical evidence remains limited. To date, no randomised clinical trials have demonstrated that vaccination of cats leads to clinically meaningful improvements in allergic disease in their owners under real-world conditions. In contrast, studies focused on immunogenicity, allergen measurements, and surrogate markers of allergenic activity rather than direct assessment of clinical outcomes in allergic individuals. Consequently, it remains uncertain to what extent reductions in biologically active Fel d 1 translate into sustained improvements in allergic symptoms, medication use, asthma control, or quality of life under real-world conditions. Well-designed prospective clinical studies in cat-allergic individuals are therefore needed to establish the true clinical effectiveness of this approach. Collectively, the available data provide compelling proof-of-concept that vaccination against Fel d 1 can reduce biologically active allergen. Whether this strategy ultimately translates into meaningful clinical benefit for cat-allergic individuals remains the key question for future research.

### Opportunities and remaining challenges

The hypocat studies provide proof-of-concept that source-directed immunological neutralisation of Fel d 1 is feasible, immunogenic, and apparently well tolerated (37, 38). Compared with repeated physical allergen-removal strategies, vaccination may offer the prospect of sustained allergen control through endogenous antibody production representing a potentially complementary approach to conventional environmental control and patient-directed therapies. Furthermore, vaccination is potentially compatible with other source-directed interventions, including dietary allergen-neutralisation approaches, grooming measures, and environmental control strategies.

Several important questions nevertheless remain unanswered. Long-term durability of antibody responses, optimal booster schedules, and real-world effectiveness in reducing symptoms among allergic individuals require further study. Equally important is the continued evaluation of feline health and welfare, particularly given the incomplete understanding of the physiological role of Fel d 1. Although no major safety concerns have been reported to date, larger studies with longer follow-up will be necessary to fully characterise the consequences of sustained Fel d 1 neutralisation. Beyond demonstrating clinical efficacy and long-term safety, successful translation into routine practice will also depend on regulatory approval, acceptance by veterinarians and cat owners, cost-effectiveness, and integration into existing allergy management strategies. Future clinical studies should therefore focus on patient-centred outcomes and determine whether vaccination enables allergic individuals to maintain contact with their cats while achieving sustained symptom control. If these challenges can be addressed, vaccination against Fel d 1 could become one component of future integrated management strategies that combine source-directed allergen reduction with patient-directed therapies. Ultimately, the success of this approach will depend not only on its biological efficacy but also on demonstrating clinically meaningful benefit for allergic individuals in well-designed prospective clinical trials.

## Physical reduction of allergen load on the cat

### Rationale for physical allergen removal

Unlike source-directed immunological approaches that seek to reduce the production or biological activity of Fel d 1, physical interventions aim to remove allergen after it has been deposited onto the fur and skin of the animal. Because Fel d 1 is transferred from saliva to the coat during grooming, the fur represents an important reservoir from which allergen is continuously released into the environment. Mechanical removal of allergen from the coat therefore has the potential to reduce environmental contamination and subsequent human exposure. Physical allergen reduction strategies are of course attractive because they are non-pharmacological, relatively inexpensive, and can be implemented wihout altering the cat's biology. Consequently, bathing, grooming, brushing, and the use of allergen-reducing wipes have frequently been recommended as adjunctive measures for cat-allergic individuals wishing to maintain contact with their pets.

### Bathing and grooming interventions

Several studies have investigated whether washing cats can reduce Fel d 1 levels on the fur and in the surroundings (54). In general, bathing results in an immediate but transient reduction of surface-associated allergen. Fel d 1 is continuously replenished through ongoing secretion and grooming behaviour. Studies have demonstrated that allergen levels may return to baseline within days or even hours following bathing (6, 23, 55), necessitating frequent repetition to maintain reduced allergen loads. Similarly, routine grooming and brushing may remove allergen-containing hair, dander, and surface contaminants from the coat. While these interventions can reduce the amount of allergen available for environmental dissemination, their effectiveness depends on grooming frequency, the amount of hair removed, and the subsequent handling of allergen-containing material. In practice, grooming performed indoors may transiently increase airborne allergen concentrations through resuspension and dispersal of Fel d 1-containing particles, particularly in the absence of adequate ventilation or allergen control measures (31).

A practical limitation of both bathing and grooming is cat compliance. Many cats experience stress during bathing procedures, which may limit long-term adherence and raise animal welfare considerations. Consequently, although these approaches can transiently reduce allergen burden, their utility as stand-alone interventions is likely limited.

### Allergen-reducing wipes and topical products

More recently, commercially available wipes, sprays, and other topical formulations have been developed to reduce amount of allergen on the cat's coat without the need for extensive bathing (4). These products are designed either to physically remove allergen-containing material or to bind and inactivate surface-associated Fel d 1. Compared with bathing, wipes may offer improved practicality and owner acceptance, allowing more frequent application with less stress to the animal. However, evidence supporting their efficacy remains limited, and few studies have evaluated clinically relevant outcomes beyond measurements of allergen levels on the fur or in household dust. As with bathing, any reduction in allergen burden is temporary because the underlying production of Fel d 1 remains unaffected.

### Clinical relevance and limitations

From a mechanistic perspective, physical allergen removal addresses only the final stage of allergen dissemination and does not change the cat's Fel d 1 production. Consequently, these interventions are inherently reactive rather than preventive. This distinguishes them from emerging immunological approaches that seek to reduce the amount of biologically active allergen before it reaches the fur or environment.

A recurring limitation of the available literature is the reliance on surrogate endpoints such as reductions in Fel d 1 concentrations on fur, in settled dust, or in airborne samples, or whether such reductions translate into clinically meaningful improvements, e.g., allergy symptoms, medication use, or quality of life. Furthermore, the magnitude and duration of allergen reduction vary considerably between studies, making comparisons difficult. Despite these limitations, physical allergen removal may have value as part of a multifaceted allergen-management strategy. For individuals who choose to keep a cat despite sensitisation, grooming, bathing, and topical allergen-reducing products may contribute to lowering cumulative exposure when combined with environmental interventions such as air filtration, regular cleaning, and restricted access to bedrooms. However, current evidence does not support these measures as a substitute for more effective allergen-reduction strategies.

## Genetic and breeding approaches

### Natural variation in Fel d 1 production

Fel d 1 production varies considerably between individual cats, providing the biological basis for genetic and breeding approaches aimed at reducing allergen production. Understanding this natural variability is therefore an essential prerequisite for developing long-term source-directed strategies. Studies have demonstrated substantial differences in allergen levels between animals, with variation observed according to sex, neutering status, age, and genetic background. Male cats generally produce higher levels of Fel d 1 than females (23, 56, 57), while castration is associated with reduced allergen production (57, 58), suggesting hormonal regulation of allergen expression. Importantly, the magnitude of variation between individual cats often exceeds the differences reported between breeds. The latter highlights the complex genetic and environmental factors that contribute to Fel d 1 production and suggests that allergenicity should be considered a quantitative rather than binary trait.

### The concept of hypoallergenic cats

The existence of “hypoallergenic” cat breeds remains a topic of considerable interest among allergic individuals. Several breeds, including Sphynx, Siberian, Bengal, Balinese, Russian Blue, Cornish Rex, and Devon Rex have been marketed as producing lower levels of allergen or causing fewer allergic symptoms (59–62) However, scientific evidence supporting the hypoallergenic status of specific breeds remains limited. While some studies have reported lower average Fel d 1 levels in selected breeds, substantial overlap exists between breeds, and no breed has been shown to be consistently allergen-free (63). Furthermore, allergen production may vary considerably among individual animals within the same breed. Consequently, the term “hypoallergenic cat” should be interpreted with caution. Current evidence suggests that selecting individual cats with lower allergen production may be more relevant than breed selection alone.

The concept of hypoallergenic cats has attracted considerable public interest and commercial attention for more than two decades. This interest reflects the substantial unmet need among cat-allergic individuals who wish to maintain contact with companion animals while minimising allergic symptoms. However, despite numerous claims regarding hypoallergenic breeds or low-allergen cats, scientific evidence supporting the existence of truly hypoallergenic cats remains limited.

### Future opportunities for genetic selection

Advances in feline genomics are creating new opportunities to better understand the biological determinants of Fel d 1 production. Identification of genetic variants associated with low-allergen phenotypes could facilitate selective breeding programs aimed at reducing allergen production while preserving desirable breed characteristics. Such approaches may ultimately prove more effective than relying on breed designation alone. Beyond conventional breeding, emerging genome-editing technologies have raised the possibility of directly modifying genes involved in Fel d 1 production. Proof-of-concept studies have demonstrated the feasibility of targeting the CH1 and CH2 genes encoding Fel d 1, highlighting the potential for future development of cats with substantially reduced allergen production (59, 62).

Despite these promising proof-of-concept studies, substantial technical and translational challenges remain before genome editing could be considered a practical strategy. Current genome-editing technologies may introduce unintended off-target genetic modifications (64, 65), and the long-term biological consequences of permanently disrupting Fel d 1 expression remain uncertain (23) and warrant careful evaluation. Because the physiological functions of Fel d 1 remain incompletely understood, it cannot yet be excluded that genetic modification could affect feline behaviour, social communication, reproduction, skin biology, or other aspects of health. Furthermore, regulatory approval of genome-edited companion animals differs substantially between countries and is likely to present significant legal and societal challenges. Ethical considerations are equally important, including questions regarding animal welfare, the acceptability of modifying companion animals primarily to benefit humans, and the broader implications of introducing genetically modified pets into society.

Although genetic approaches remain at an early stage of development, they offer the prospect of permanent and lifelong reduction of allergen production. Future research should focus on elucidating the biological function of Fel d 1, identifying genetic determinants of allergen expression, and evaluating the safety, efficacy, and ethical implications of breeding or engineering cats with reduced allergenic potential.

## Emerging concepts: from single interventions to integrated allergen management

### Dietary neutralisation of Fel d 1

A novel approach to source-directed allergen control involves the neutralisation of Fel d 1 within the cat before environmental dissemination occurs. The most extensively studied example is the incorporation of anti-Fel d 1 immunoglobulin Y (IgY) antibodies derived from chicken eggs into cat food (66–68) Following ingestion, these antibodies bind Fel d 1 present in feline saliva. Because saliva is the primary source of Fel d 1 deposited onto the fur during grooming, this strategy aims to reduce the amount of biologically active allergen subsequently released into the environment. Importantly, the approach does not seek to suppress Fel d 1 production but rather to neutralise allergen activity before human exposure occurs. Several studies have reported reductions in active Fel d 1 levels on the fur and in the environment following dietary supplementation with anti-Fel d 1 IgY (67, 68). While safety has been shown in independent studies (69), evidence demonstrating direct clinical benefit in allergic individuals remains limited, and long-term effectiveness under real-world conditions requires further investigation.

### Passive immunological approaches

The success of vaccination and dietary antibody-based interventions highlights the broader potential of immunological allergen-neutralisation strategies. In principle, passive administration of Fel d 1-specific antibodies could provide an alternative means of reducing allergen activity without requiring the induction of an endogenous immune response in the cat. However, compared with active vaccination, passive immunization faces several practical challenges. Administered antibodies are gradually cleared from circulation and tissues, necessitating repeated treatment to maintain efficacy. Depending on the formulation and route of administration, dosing intervals would likely range from weeks to months. Furthermore, the large-scale production of monoclonal or polyclonal antibodies remains costly, raising concerns regarding affordability and long-term implementation in companion animals.

Additional challenges include achieving sufficient antibody concentrations at the sites of Fel d 1 production and secretion, as well as ensuring sustained neutralisation of newly synthesised allergen. Consequently, although passive immunisation represents an intriguing conceptual approach, its practical application may be limited compared with active vaccination strategies that enable the cat to continuously generate anti-Fel d 1 antibodies following immunisation. Given the demonstrated feasibility of active Fel d 1 vaccination, passive immunisation may ultimately find greater utility as a research tool than as a practical long-term strategy for routine allergen management.

### Combination strategies and future clinical development

Future management of cat allergy is unlikely to rely on a single intervention. Instead, combinations of complementary approaches may provide the greatest reduction in allergen exposure. For example, vaccination or dietary allergen neutralisation could be combined with environmental measures and targeted grooming practices to achieve additive effects. Such integrated strategies represent a shift away from traditional avoidance-based recommendations and toward proactive management of allergen production and dissemination. As the field matures, future studies should focus not only on reductions in Fel d 1 concentrations but also on clinically meaningful outcomes, including symptom control, quality of life, and the ability of allergic individuals to maintain contact with companion animals.

The future of cat allergy management may not depend on identifying a single highly effective intervention but rather on combining complementary approaches that reduce allergen production, allergen shedding, environmental burden, and host sensitivity. Table 2 shows a comparison of the above-discussed, source-directed allergen-reduction measures.

**Table 2:** Comparison of source-directed allergen-reduction strategies. The interventions listed differ substantially in their evidence base, practicality, and implications for animal welfare. Direct comparisons between strategies should therefore be interpreted with caution.

Strategy	Mechanism	Stage targeted	Expected duration of effect	Advantages	Limitations	Development stage	Levels of evidence
Vaccination	Anti-Fel d 1 IgG/IgA	Allergen production and allergen activity	Months to years (with booster vaccination)	Long-lasting effect; source-directed; potentially reduces biologically active allergen before environmental dissemination	Limited clinical outcome data; requires vaccination and booster schedule; long-term consequences of Fel d 1 neutralization remain incompletely understood	Pre-clinical, veterinary field studies	Moderate
Dietary IgY	Anti-Fel d 1 IgY	Allergen activity and shedding	Months (continuous feeding required)	Non-invasive; easy implementation; compatible with other interventions	Requires continuous feeding; limited human clinical outcome data; ongoing cost	Commercially available, translational studies	Moderate
Grooming, wipes	Physical Fel d 1 removal	Allergen on fur	Days to weeks	Simple; non-pharmacological; can be combined with other measures	Transient effect; repeated application required; variable cat acceptance	Established	Low-moderate
Bathing	Physical Fel d 1 removal	Allergen on fur	Days to weeks	Immediate reduction of surface allergen burden	Short-lived effect; poor compliance in many cats; potential stress for the animal	Established	Low-moderate
Environmental control	Removal of allergen from indoor environment (e.g., cleaning, HEPA filtration)	Environmental allergen burden	Continuous while maintained	Applicable regardless of allergen source; may complement source-directed interventions	Does not reduce allergen production; labour-intensive; difficult to achieve complete allergen removal	Partly established	Moderate
Breeding, genetics	Reduced Fel d 1 production	Allergen production	Lifelong	Potentially permanent reduction in allergen production	Limited evidence; substantial variability between individuals; ethical, welfare, and biological considerations	Experimental	Low

## Conclusions and future perspectives

Cat allergy remains a major cause of allergic rhinitis and asthma worldwide, and allergen avoidance is often challenging because of the ubiquitous distribution of Fel d 1 and the strong bond between humans and their companion animals. While traditional management has focused primarily on treating allergic individuals through pharmacotherapy, AIT, and environmental control measures, emerging source-directed approaches seek to reduce allergen exposure at its origin.

Vaccination strategies that induce anti-Fel d 1 antibodies, dietary approaches that neutralise allergen activity, physical methods that reduce allergen burden on the coat, and genetic approaches aimed at lowering allergen production collectively represent a paradigm shift from managing the consequences of allergen exposure to controlling allergen production and dissemination at its source. These interventions should not be viewed as alternatives to patient-directed therapies but as complementary components of an integrated management strategy. Future management of pet allergy will likely combine source-directed interventions with improved patient-directed therapies, including safer and more effective forms of AIT, to reduce both allergen exposure and host sensitivity.

Future developments should also consider the perspectives of pet owners and their families, for whom companion animals provide important emotional, psychological, and social benefits. Equally important are considerations of animal welfare. Unlike most veterinary interventions, many source-directed allergen-control strategies are designed primarily to benefit allergic individuals rather than the animals themselves. Consequently, their development and implementation should be guided not only by efficacy but also by careful evaluation of their impact on feline health, behaviour, and well-being. Interventions that are safe, minimally invasive, and compatible with normal feline physiology and behaviour are likely to achieve greater acceptance among veterinarians, pet owners, and society at large. In some cases, source-directed interventions may ultimately benefit both humans and animals by reducing the need for pet relinquishment or euthanasia due to allergy-related concerns.

Ultimately, the goal of pet allergy management is not necessarily the complete elimination of allergen exposure but rather enabling allergic individuals and companion animals to coexist with minimal disease burden while preserving quality of life, the human-animal bond, and animal welfare. Continued advances in source-directed allergen control, together with improvements in patient-directed therapies, may help transform this goal into a practical reality. Finally, future management of cat allergy will likely require integration of source-directed allergen reduction with patient-directed therapies while also considering broader biological factors, including epithelial barrier function and environmental influences on allergic disease.
